# Going beyond Clustering in MD Trajectory Analysis: An Application to Villin Headpiece Folding

**DOI:** 10.1371/journal.pone.0009890

**Published:** 2010-04-15

**Authors:** Aruna Rajan, Lydia Freddolino, Klaus Schulten

**Affiliations:** 1 Department of Physics, University of Illinois, Urbana-Champaign, Illinois, United States of America; 2 Beckman Institute for Advanced Science and Technology, University of Illinois, Urbana-Champaign, Illinois, United States of America; Monash University, Australia

## Abstract

Recent advances in computing technology have enabled microsecond long all-atom molecular dynamics (MD) simulations of biological systems. Methods that can distill the salient features of such large trajectories are now urgently needed. Conventional clustering methods used to analyze MD trajectories suffer from various setbacks, namely (i) they are not data driven, (ii) they are unstable to noise and changes in cut-off parameters such as cluster radius and cluster number, and (iii) they do not reduce the dimensionality of the trajectories, and hence are unsuitable for finding collective coordinates. We advocate the application of principal component analysis (PCA) and a non-metric multidimensional scaling (nMDS) method to reduce MD trajectories and overcome the drawbacks of clustering. To illustrate the superiority of nMDS over other methods in reducing data and reproducing salient features, we analyze three complete villin headpiece folding trajectories. Our analysis suggests that the folding process of the villin headpiece is structurally heterogeneous.

## Introduction

Molecular Dynamics (MD) simulations are frequently used today to study protein folding. The greatest challenges that MD simulations of protein folding face are those of timescale and accuracy. The currently known fastest folding proteins fold over 0.7–1.0 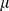
s [1]. There is a hypothesized limit of around (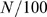) 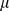
s for the folding timescale of an 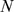 residue protein [2]. Only recently has technological progress enabled atomistic MD simulations to probe microsecond timescales regularly [3]–[5].

One of the most commonly studied fast folding proteins is the villin headpiece, a 35 residue actin-binding domain, which folds into a three helix bundle with a hydrophobic core in about 4.5 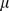
s [6]. In 1998, Duan and Kollman simulated the villin headpiece in what had been the longest simulation (of 1 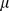
s) until then [7]. Complete explicit solvent MD folding trajectories for the villin headpiece were recently obtained by Freddolino and Schulten [6]. The protein folded to its native state, starting from a completely unfolded state in three different trajectories of 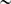
6 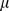
s each, and continued to be stable for more than 1 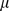
s after folding. Such a folding trajectory contains millions of frames (each frame being one snapshot in time of all of the protein's atomic coordinates) and in order to obtain a qualitative picture of the folding process and to find collective coordinates of folding, if any, it is important to obtain reduced representations of these trajectories.

Conventional clustering algorithms used to reduce MD trajectories [8]–[10] require specification of the number of clusters or a cluster radius, making the clustering artificial, that is (i) inter-cluster relationships are not taken into account and (ii) the clusters are unstable against small changes in cutoff parameters and noise in the data. When simple cut-off based clustering was applied to villin folding trajectories using the program GROMACS [11], varying the cluster radius in a range of 2 to 6 Å was found to shift the cluster centers. Some of the clusters that were maximally occupied when the trajectory was clustered with a smaller cutoff, merged into larger clusters when the cutoff was changed by 1 Å. In addition, the clustering was not stable when the trajectories were binned more coarsely or finely in time by up to five times. While such clustering analyses may be acceptable for qualitatively visualizing MD trajectories, their use to study the number of structural transitions present in the trajectories and perform free energy calculations such as in [12], may lead to serious artifacts. Furthermore, partitions generated by clustering are generally validated by visual inspection of the structures returned as cluster centers. Since little is known about protein dynamics en-route to folding, visual inspection may not be a reliable way of validating clustering techniques applied to MD simulations of protein folding.

Various rigorous cluster validation methods, which take into account inter-cluster relationships have been developed in the field of bioinformatics [13]. It can nevertheless be quite difficult to choose the necessary and sufficient set of validation techniques for MD trajectories without prior knowledge of the structural processes underlying folding. An additional goal of MD simulations of folding processes is to find collective coordinates. Clustering does not yield itself to such analysis. There is clearly a need to go beyond clustering to analyze MD folding trajectories. In this paper, we report application of data reduction methods to analyze villin headpiece folding trajectories. Our methods can be used for reducing any large MD trajectory to obtain salient features.

The most widely used technique to obtain collective coordinates from folding trajectories and experiments is principal component analysis (PCA) [14]–[16]. However, apart from having other well known drawbacks [17], PCA is unable to achieve sufficient data compression when the data are nonlinearly correlated. Our trajectories reside in a high dimensional space as every snapshot has information about all atomic coordinates. However, not all coordinates are important to folding; many coordinates are likely to be nonlinearly correlated and, thus, if viewed in the correct coordinate space, the folding trajectories might lie in some lower dimensional space. The extraction of a correct reduced basis has been the goal of a variety of dimensional reduction methods. Apart from PCA, which was first applied in 1992 [14] to the study of protein folding, other multidimensional scaling methods have been applied to protein folding trajectories [18], [19]. We have adapted a *non-metric multidimensional scaling method (nMDS)* for our analysis [20]–[24]. nMDS is a completely data driven scheme and in our experience its performance is superior to other methods of its class (except perhaps in terms of computational requirements). The dimensionality of the representation is reduced by nMDS while preserving the inter-relationships of the data points (described in detail in the following section). There is no standard recipe for interpreting the axes obtained after nMDS embedding. The situation is not very different in PCA, where, although the axes are known mathematically, it may be hard to find a simple interpretation for them, especially if the original trajectories reside in a high dimensional space. Often reduced axes have to be inferred by visual inspection of the projected data. There are nonlinear versions of PCA that may be used for dimensionality reduction, similar to Coifman et al's diffusion maps [25], but these versions of PCA differ from nMDS in that they are not truly data driven and depend on the choice of kernel used. By appropriately selecting a kernel, reasonable results may be achieved. However, it is hard to find a reasonable kernel without *a priori* information about the data set. As we know very little about the differences between structures enroute to folding, we choose to work with a metric free multidimensional scaling method.In the following sections, we discuss the nMDS method and the results obtained from applying PCA and nMDS to our trajectories.

## Methods

**Figure 1:**
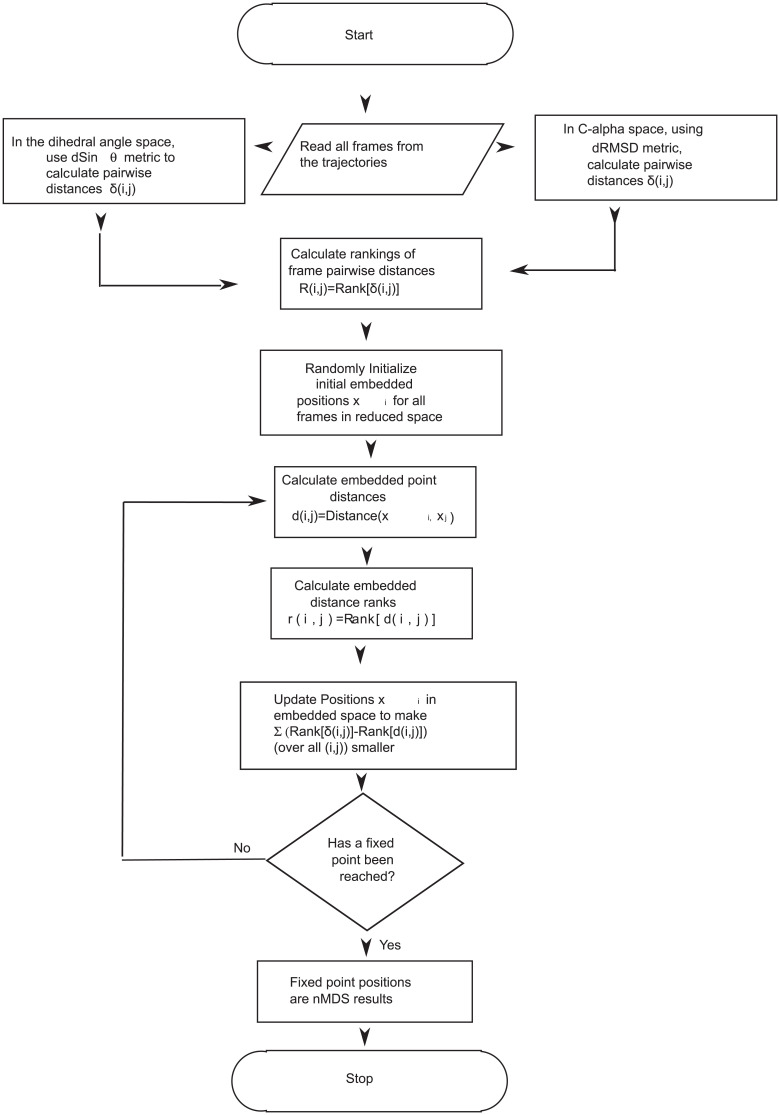
nMDS algorithm. Flowchart describing data embedding to a reduced space from a higher dimensional input space (dihedral angles or cartesian coordinates).

In this section, we explain the implementation of nMDS. nMDS is an unsupervised data geometrization method placing 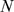 points representing the objects under study (in our case, the 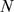 frames of an MD trajectory), in a certain metric space 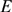 (explained for our particular case later), such that the pairwise distances 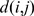 of the points in 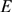 have consistency with the pairwise dissimilarities 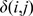 of the corresponding objects in the input data [20]–[23], [26], [27]. More precisely, nMDS tries to ensure that if 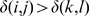, then 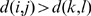 for all 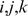 and 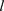 denoting objects being analyzed. It is considered non-metric because, strictly speaking, the 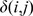 values need not be known; only their order relationships need to be known, i.e., whether 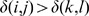 holds or not. If we have a reasonable number (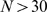) of points, this condition is typically strong enough to ensure a unique geometrical pattern for good data [28]. There are many possible implementations of an nMDS algorithm outlined above. We use an algorithm that has been successfully applied by Taguchi and Oono [24], [29]–[31] to large sets of gene expression time series data to unravel relational patterns among genes. A flowchart explaining the application of nMDS to MD trajectory data is shown in Fig. 1.

If the pairwise dissimilarity 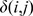 has ranking 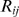 in the set of all the available dissimilarities, and 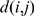 has ranking 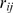 in the set of all the pairwise distances of the points in 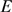, the points in 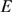 are positioned to minimize
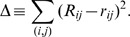
The minimum of this is achieved when 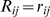 for all 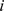 and 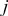, *i.e.*, when the pairwise rankings in the original data and those in the embedded space exactly match. This is achieved through an over-damped dynamics driven by the ranking mismatch. The updating scheme used is:
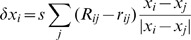
where the positions of the points in 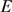 are given by 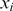 and 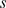 is an appropriately small number to make the relaxation dynamics stable. The 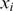 are initially chosen randomly, and the positions are updated using the rule above until a fixed point is reached. It should be clear that the desired minimum of 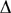 does correspond to fixed point of the dynamics. Like all nonlinear-optimization methods, there is a risk of getting trapped in a fixed point that is a local minimum, although in practice the dependence on initial condition seems to be weak compared to the dependence found for other methods.

If additional structures are added to a trajectory, which are smaller in number compared to the already embedded structures in the projected space, then we may construct a new mapping for the trajectory by maximally respecting the old configuration in the projected space. The scheme is similar to Scheme S outlined in Rajaram et al. [32], where each point is updated around its position in the old configuration in the projected space.

Comparison of the embedding done by nMDS to that produced by PCA is done in our case to check if nMDS achieves a reasonable embedding.

## Results and Discussion

We now illustrate the usefulness of PCA and nMDS in finding reduced representations for large MD trajectories using three complete villin headpiece folding trajectories. In order to apply PCA/nMDS, we chose to work with backbone dihedral angles (to study local structure formation) and 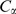 coordinates (to study tertiary structure formation). Below, we describe our findings in each of these input spaces. Note that a variety of metrics are used in our analysis (described ahead), different from the metric used in [6] and also our trajectories are binned more coarsely than in [6].

### Dihedral angle space

**Figure 2:**
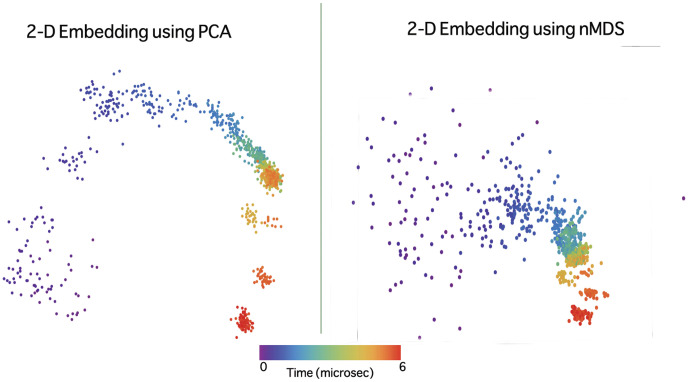
Stability of nMDS to bin size. nMDS embedding (in 2D) of villin trajectory 1 data in the dihedral angle space is shown when the binning time was varied between 1 ns and 30 ns. The patterns in the projected space remained stable with change in binning time.

The trajectories were binned at every 6 ns and every resultant snapshot/frame was read in as a 70-dimensional vector (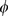/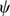 angles for the 35 residues) to obtain about 1000 vectors for each trajectory. To do PCA in the dihedral angle space, similar to the version outlined in Wang and Brüschweiler [33], we take the covariance matrix, whose elements are given by 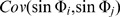 (where 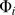 and 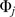 are the corresponding dihedral angles of a residue in two different structures i and j). On applying this linear PCA, we found that in all three trajectories, 90 percent of the total amplitude of fluctuations was captured by the 6 largest amplitude modes. A nonlinear dimension reduction method is hence sure to yield good compression for up to 3 or 4 dimensions. We applied nMDS to all trajectory data using a Euclidean distance 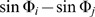, (where 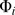 and 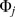 are as defined before) in the 70-dimensional input space as a metric to assign dissimilarities. It was found that a two dimensional Euclidean space was enough to capture the variations in the data (this was checked by applying PCA to the embedded results obtained from nMDS reduction to 2, 3, 4 and 5 dimensions as described in [29]). The idea is as follows: Suppose we use nMDS to embed identical data into 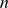-dimensional and 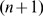-dimensional spaces. Using the embedded results and applying PCA to them, we can construct principal axes. Then, we study the correlation of the principal axes obtained thus. Usually, the first 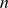 principal axes of the 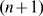 dimensional embedding result have high correlation coefficients with the 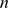 principal axes of the 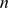-dimensional embedding result. If the correlation between the 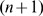 th axes of an 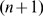 dimensional embedding with that of the 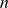 axes of an 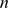 dimensional embedding is very small, then we may say that the 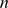-dimensional reduced space captures the main features in the data. In Tables 1 and 2, we show the application of this method to 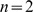 in the dihedral angle space for all trajectories. As can be seen from the table, we may conclude that 2D is necessary and sufficient to capture the main features in the data. We also checked that the reduced representation in two dimensions remained unaffected by binning our trajectories by up to five times more coarsely or finely in time (see Fig. 2). Hence, nMDS proves to be stable when used to view trajectories at different time scales.

**Table 1:** Correlation coefficients between 2D and 3D axes obtained by applying PCA to nMDS results on all villin trajectories in the dihedral angle space.

Traj 1	3DI	3DII	3DIII
2DI	0.982	0.012	0.002
2DII	0.012	0.975	0.005

Traj 2	3DI	3DII	3DIII
2DI	0.992	0.009	0.001
2DII	0.011	0.989	0.001

Traj 3	3DI	3DII	3DIII
2DI	0.965	0.018	0.003
2DII	0.03	0.934	0.01

**Table 2:** Correlation coefficients between 2D and 1D axes obtained by applying PCA to nMDS results on all villin trajectories in the dihedral angle space.

Traj 1	2DI	2DII
1D	0.632	0.294

Traj 2	2DI	2DII
1D	0.812	0.178

Traj 3	2DI	2DII
1D	0.56	0.32

**Figure 3:**
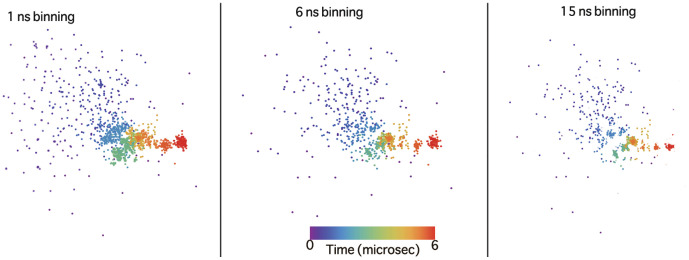
PCA and nMDS embedded representation of trajectory 1 applied to dihedral angle space. Two panels showing the embedding of trajectory 1 from the dihedral angle space to a 2D space obtained by PCA (left) and nMDS (right). It may seem that PCA separates data more clearly into clusters, however we must not read too much into PCA results. The first two axes suffice to embed all the data in nMDS, whereas with PCA, they capture only 50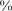 of the total amplitude fluctuation in the data. Both PCA and nMDS do well to separate the structures into clusters and this shows that when a nonlinear method like nMDS is used to reduce representation, PCA may be used to construct linear maps from PCA axes to nMDS axes. However, we do not have sufficient data to do this.

Figure 3 shows the embedded space representation for trajectory 1 obtained by PCA and nMDS. Note that if some structures lie closer to each other than the other structures in the projected space, we call them a “cluster” for the purpose of qualitative analysis. Although it seems like PCA produces better clusters from Fig. 3, it should be noted that these clusters are illusory. The first two principal components only capture 50% of the fluctuations in the data and, moreover, when the number of data points were increased to over 2000 by binning more finely in time, PCA was unable to project the partially folded initial (occurring between 500 to 900 ns) states clearly. Many of these initial states lay close to the native cluster after PCA was applied. PCA results are hence unstable to finer binning in time. nMDS proved to be robust in preserving inter-relationships between structures, although it is computationally expensive when the data size was increased. Hence, we chose to work with nMDS embedding to analyze the villin folding trajectories. However, PCA still proves to be a computationally cheaper first look at the trajectories and nMDS can be used to find further structure in the data set if needed.

**Figure 4:**
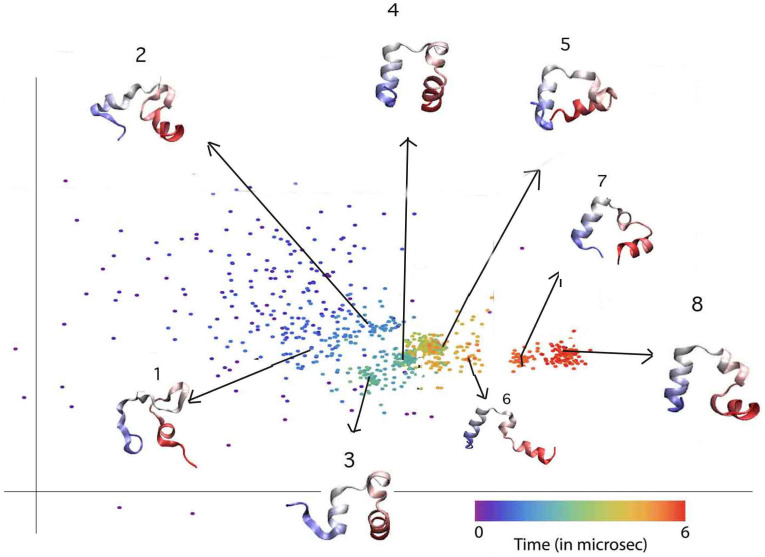
Reduced representation of Trajectory 1. In the embedded 2D space (structures numbered chronologically). Helix 1 and 3 (in blue and red resp.) form very quickly, but helix 2 (in white) forms only towards the end when helix 1 adopts the correct orientation with respect to the rest of the structure. Each representative structure is superimposed over the native state to show folding.

**Figure 5:**
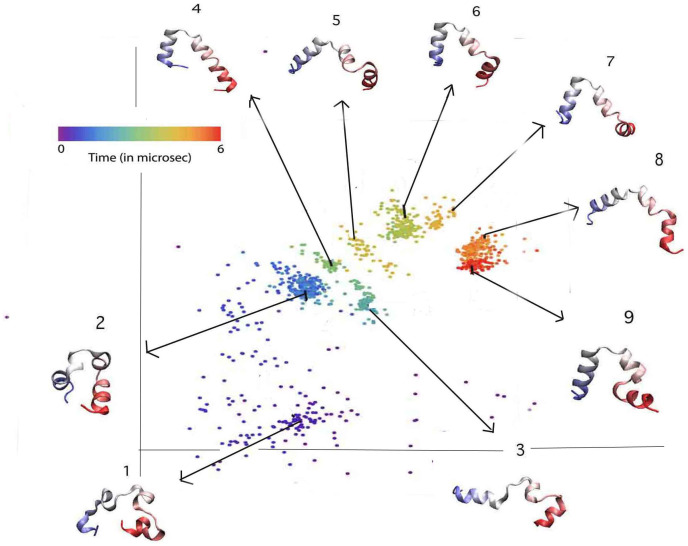
Reduced representation of Trajectory 2. In the embedded 2D space (structures numbered chronologically). All three helices form very quickly but their relative orientations are incorrect. Parts of these helices then dissociate, form non native contacts and finally rearrange to reach the correct structure.

**Figure 6:**
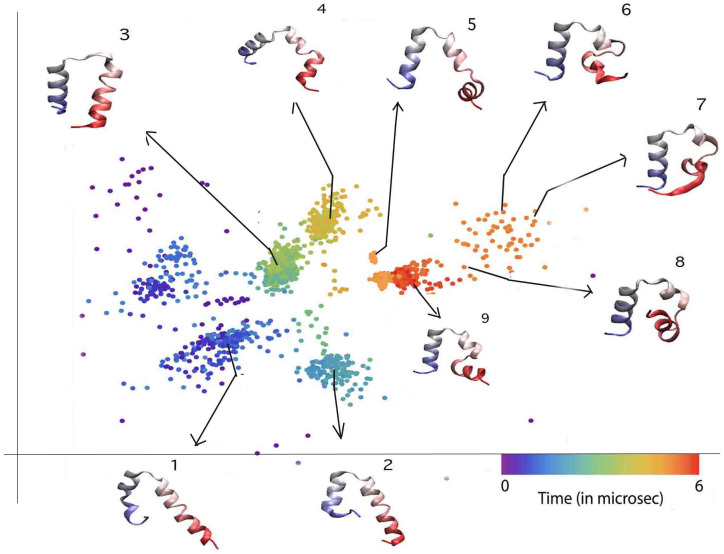
Reduced representation of Trajectory 3. In the embedded 2D space (structures numbered chronologically). A two-helix conformation with helix 2 and helix 1 joined is very stable for the first 3 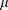
s; the protein then dissociates these helices and adopts the correct tertiary structure.

We picked representative structures from the densely occupied portions of the reduced conformational space (in 2D) to obtain a reduced representation for the trajectories (see Figs. 4, 5 and 6). nMDS results show that the conformational space explored by the protein narrowed with time as expected. In all three trajectories, the protein initially explores conformational space in what seems like random motion after which the secondary structure elements begin to form. In Trajectory 2 and 3, all three helices form within the first 400 ns. In Trajectory 1, it takes up to 1 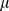
s for helix 1 and helix 3 to form and helix 2 forms only in the last microsecond. We can see natural clustering in all cases, which implies that there are many fairly well defined metastable states. When simple clustering was used, a large number of clusters (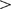
100) were found, but nMDS and PCA show clearly that there are not more than 5 or 6 distinctly densely populated regions in the explored phase space. This shows again that simple clustering may unnecessarily split similar structures into different clusters.

**Figure 7:**
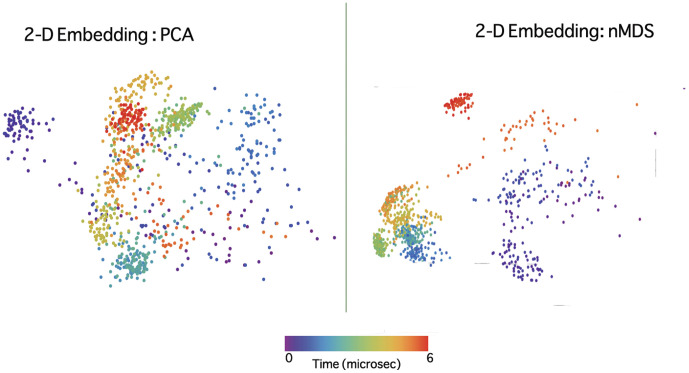
Separation of the trajectories in reduced dihedral angle space. Along one of the axes, there are many crossing points beteween the trajectories. The crossing points were found to correspond to similar secondary structure elements forming, i.e. formation of helix 1 and 3. Along the other axis however, trajectory 1 is separated until it reaches the cluster containing the native state. Trajectories 2 and 3 meet at the two-helix states. The double helix (DH) and flipped (F) states are marked for each trajectory in the figure. Note that the flipped state of trajectory 1 is different from that of trajectories 2 and 3.

In order to find collective coordinates for villin folding, we must ask how similar the three trajectories are. Is there a structure or a cluster of structures that occur in all three trajectories? To answer this, we must study how close to each other the data points across three trajectories lie in the reduced (projected) space. We applied nMDS to data from all three trajectories together after removing noise (dihedral angles of floppy residues: residue numbers 1–3, 11–12 and 32–35) and found that the structures from different trajectories clustered very differently in the 2D projected space, except for a few similarities. We found that along one of the axes in the 2D projection, the trajectories met at a few points, which on visual examination showed that helix 1 and helix 3 were completely formed for those data points in all trajectories. However, along the second dimension, the trajectories were still slightly separated (the separation could be due to the difference in conformation of the residues forming helix 2) except for around the native state where they met again (Fig 7). Notice that in our reduced space separation implies real distinction of conformations. Needless to say, coincidence does not necessarily imply agreement of conformations. Trajectories 2 and 3 had more similarities with each other than with Trajectory 1 in the second projected axis. It was found that both Trajectories 2 and 3 had two-helix structures similar to that shown in Fig. 7. Although in trajectory 2, these structures occurred transiently, in trajectory 3 they seemed to be very stable and lasted for up to 3 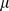
s. Some of the qualitative features we distill from nMDS and PCA closely reproduce those obtained from careful visual inspection by Freddolino and Schulten [6].

nMDS results thus show that the path to the native state along the dihedral angle coordinates differs qualitatively for all trajectories, although a few trivial similarities like the rapid formation of helix 1 and 3 exist. One axis may be interpreted as pertaining to formation of helices 1 and 3 and the other pertaining to local structure of the residues forming helix 2 and the coil regions. Since PCA results and nMDS results looked very similar for the dihedral angle space, if enough trajectories become available in the future, it might be possible to construct a map between the PCA axes and the nMDS axes. The axes obtained by PCA are known linear combinations of the input dimensions. By constructing a simple map (for e.g., quadratic or some power series) from the coordinates of the projected data in the PCA-reduced space to the corresponding coordinates in nMDS-reduced space, we can attempt to reconstruct the nMDS coordinates. However, when we attempted to map PCA axes to the corresponding nMDS axes using three trajectories, the data was insufficient for a clear interpretation to emerge. This is perhaps because we have only three trajectories showing large heterogeneity. Empirical evidence from the use of nMDS in bioinformatics suggests that if we had about 30 trajectories, it is likely that such mappings may become statistically possible [30].

In order to understand the tertiary rearrangement in folding, we need to look at the trajectories in cartesian coordinate space. After that, we attempt to combine both dihedral angle and cartesian coordinates before applying PCA/nMDS to obtain a better picture of the folding process.

### Cartesian coordinate space

**Figure 8:**
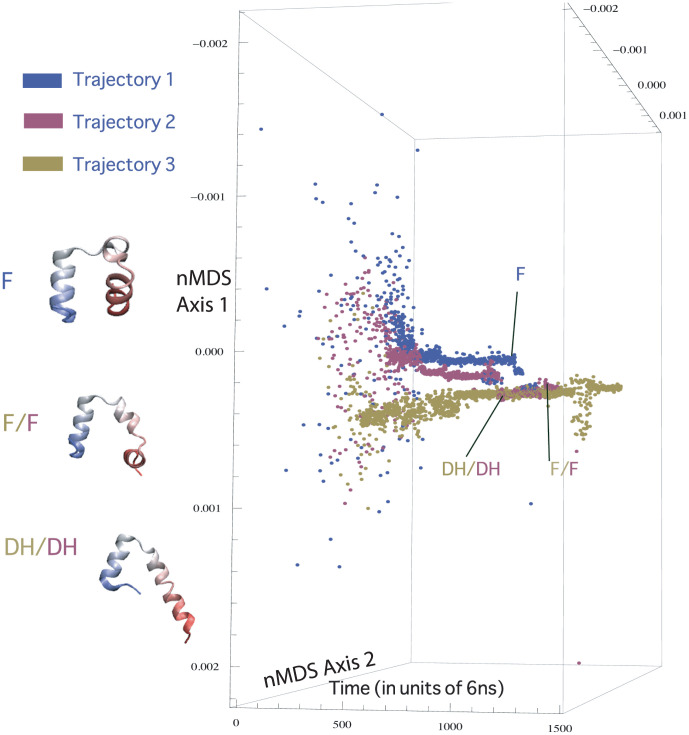
PCA and nMDS embedded representation of Trajectory 1 applied to internal coordinate space. Two panels showing the embedding of trajectory 1 from the internal coordinate space (described in the paper) to a 2D space obtained by PCA (left) and nMDS (right). PCA results were not stable when binning time was reduced, nMDS was found to be more stable. Additionally, the first two PCA axes capture only 40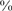 of the total amplitude fluctuation in the data.

We chose an internal coordinate system (described below) for each trajectory (similar to that used in [6] with the gromos [10] method in GROMACS [11] program for clustering) to apply dimension reduction. Suppose that there are 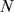 frames in the trajectory (or that the trajectory is divided into 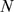 equally spaced snapshots). Then, let us construct a symmetric matrix 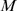 defined through 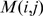

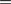
distance (RMSD) between frame 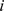 and frame 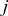. Now, each of the rows of this matrix is a vector and nMDS is applied to these 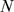 vectors. The matrix was constructed by computing the RMSD between all heavy atoms across frames after discarding some of the initial unfolded state frames and aligning all the frames (by appropriate rotations and translations). Each trajectory (binned at 6 ns) hence consisted of about 1000 vectors of 1000 dimensions each. PCA applied to these vectors again showed that the largest 6 modes captured 90 percent of the total amplitude of all the modes and hence, dimension reduction could be applied in this coordinate space. In this coordinate space, PCA did not do as well as nMDS. It is likely that the correlation between cartesian coordinates of heavy atoms across frames is nonlinear, whereas the backbone dihedral angles are probably not correlated in any significantly nonlinear way for nMDS to have a clear advantage. A comparison of PCA and nMDS 2D embedding obtained for trajectory 1, when applied to the internalised coordinate system, is shown in Figure 8.

We found that two dimensions were enough to represent the data after applying nMDS. All three trajectories again showed completely different structures in the reduced 3-D space. While in trajectory 2, rapid hydrophobic collapse led to structures similar to Structure 3 in Fig. 4 to be stable over 1–2 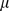
s, in trajectory 3, a two-helix structure as shown in Fig. 7 was the most stable. In all three trajectories, a similar transition referred to as a “flipping transition” in [6] happened towards the last 500 ns prior to folding. The flipping transition involved the reversal of helix 1 (flipping from pointing into the page to out of the page, with the page aligned along the plane formed by helix 2 and helix 3) [6]. The structure before helix 1 flipped into the correct native conformation will be called the flipped state in our discussion.

**Figure 9:**
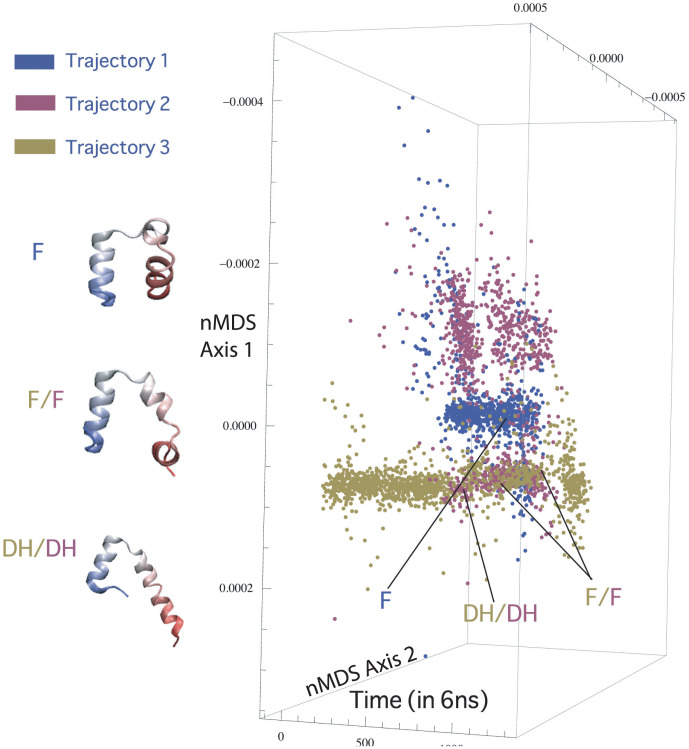
Separation of the trajectories in reduced 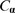 contact distances space. The points of meeting for trajectory 2 and 3 are a series of two-helix conformations (labelled as DH) and the flipped state (F). Trajectory 1 does not meet the other two trajectories except towards the last 500 ns when the protein is nearly folded. No obvious interpretation of the axes emerged on visual inspection, the trajectories showed more marked difference in the parts of projected space they explored.

Does the flipping transition occur similarly in all three trajectories? In order to answer this question, we chose 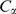 coordinates of only five of the residues (residues: 5, 8, 15, 23 and 27) forming the three helices and used the contact distances between them, together with the turning angle of helix 1 (residues 5 and 8) about the plane formed by helix 2 and 3. We now apply nMDS to all three trajectories using the contact distances described above. Two axes were found to be sufficient to represent the data and we found that the trajectories explored different portions of the projected space and met only close to the native state (Fig. 9). On visual inspection, no clear interpretation of the projected axes emerged but the points of meeting for trajectory 2 and 3 showed a series of two-helix conformations and a common flipped conformation (marked on Fig. 9). Trajectory 1 only explored transiently some of the structures that were common to Trajectory 2, and a two-helix state never occurred. The flipped state was found to be different in Trajectory 1 as compared to that of Trajectories 2 and 3 (Fig. 9) in that the second helix was formed only after the flipping happened in Trajectory 1. In Trajectories 2 and 3, helix 2 and 3 dissociated from the two-helix state described before and the protein quickly locked itself in the correct tertiary state after helix 1 flopped around exploring various non-native conformations. Although the flipped structure occurred in all three trajectories (and was slightly different in trajectory 1 structurally compared to trajectories 2 and 3 as explained above), the flipping transition was observed to occur through a different series of steps in all three trajectories. We cannot determine from the present data whether the inter-trajectory differences in the path followed during the flipping transition are due to the presence of a few distinct options, or because the intermediate is so disordered (due to the lack of tertiary contacts) that its motion is essentially diffusive until the secondary structure elements reform contacts with each other.

When both 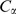 coordinates and dihedral angle coordinates were used to apply nMDS reduction, the resultant representation was dominated by the 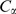 coordinate values and no new similarities between the trajectories emerged. This shows that the similarities in local structure formation are trivial and the global folding path is very different for all trajectories. Dihedral angles may hence not be good candidates for collective coordinates of small proteins such as villin headpiece. A more rigorous method to detect commonalities, ICS Survey [30] was also used in the combined 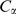 coordinates - dihedral angle space, but no interesting commonalities were found. nMDS was always used with a Euclidean metric in our results reported in this paper. However, the Euclidean metric may not be suitable to the study of all proteins/biological systems. In certain systems, it might be wiser to design more intuitive metrics that separate visually different structures even if their RMSDs are close. We also used a Hamming distance metric to rank the frames in our trajectories before applying nMDS, but this did not yield any new information. It is likely that the folding pathways are structurally heterogeneous and there are not many significant intermediates that new metrics may find. However, if larger proteins are studied, it would be desirable to design metrics that can distinguish topologically distinct structures that may lie close together if viewed in dRMSD space alone.

The most notable common feature found across trajectories using nMDS on all input spaces was the competition between local and global structure formation. If the protein formed all three helices very early like in trajectories 2 and 3, it spent a long time exploring non-native two-helix or collapsed conformations before dissociating and locking into the correct global structure. However, small changes in folding time such as this are not significant for small proteins. To understand the competition between global arrangement and local structure formation, it is important to study folding trajectories of larger proteins. To this end, we need at least four orders of magnitude faster computational speed.

In conclusion, we have shown that nMDS can achieve high compression of MD data while preserving the salient features of the underlying trajectories. We also showed that PCA is a good tool that can be applied to the data as a first step to check for any structure present in the projected data. Our analysis has convincingly been able to pick out similarities and distinguishing features of different MD folding trajectories better than any cut-off dependent clustering method. While conventional clustering methods produced unstable (to cutoff parameter changes/coarser binning in time) clusters, nMDS produced a stable representation of all trajectories showing densely populated regions of the (reduced) phase space clearly. Also, unlike tight clustering which produces a large number of clusters whose interrelationships are not known, nMDS gives a clear picture of the relationships between data points across time. Investigations of villin headpiece folding using nMDS have shown that the three villin trajectories analyzed here explore significantly different portions of the conformational space barring a few similarities such as rapid formation of most secondary structure elements and a similar flipping transition towards the end of the folding process. Many recent findings support the view of multiple routes to protein folding [34], [35]. Proteins with heterogeneity in the folding pathways may have been evolutionarily selected so that folding is ensured under very different conditions possible in the cell [35].

Much work still remains to be done with nMDS. While the compression achieved is certainly very high, nMDS is a computationally costly method and PCA may be a cheaper alternative if sufficient compression can be achieved using linear PCA. The interpretation of axes obtained after nMDS is still very dependent on visual inspection of embedded space data and perhaps some attempts can be made to construct linear maps between axes obtained by nMDS and those obtained by PCA. However for such mappings to be statistically meaningful, one will need to work with at least about 30 trajectories. It can be expected that with the advance of technology, a large number of folding trajectories may soon become available and nMDS can prove to be a robust method to find collective coordinates for description of folding processes. Although we have illustrated our case for protein folding trajectories, nMDS should be able to reduce any MD trajectories effectively.
